# First-Generation Antihistamine Use in Geriatric Emergency Department Patients: Retrospective Review

**DOI:** 10.5811/westjem.47491

**Published:** 2025-12-31

**Authors:** Emily Killen, Michael Cusumano, Zidong Zhang, Richard Newman, Jamie Voigtmann, Angela M. Sanford, Cindy C. Bitter

**Affiliations:** * Saint Louis University School of Medicine, St. Louis, Missouri; † Saint Louis University School of Medicine, Advanced Health Data Institute, St. Louis, Missouri; ‡ Saint Louis University School of Medicine, Division of Emergency Medicine, St. Louis, Missouri; § Saint Louis University Hospital, Department of Pharmacy, St. Louis, Missouri; || Saint Louis University School of Medicine, Division of Geriatric Medicine, St. Louis, Missouri

## Abstract

**Introduction:**

First-generation antihistamines are frequently used in the emergency department (ED) but are discouraged in older adults due to increased adverse drug effects. Whether concerns about adverse drug effects apply to the ED is uncertain, as ED-specific data are limited, and risks with single-dose administration may differ from risks with chronic use. In this study we assessed frequency of use, adverse drug effects, and indications of first-generation antihistamines administered to older adults during ED visits.

**Methods:**

This retrospective cohort study identified adults ≥ 65 years of age who received first-generation antihistamines from January 1–December 31, 2022 in the ED at a single, urban, academic medical center. Abstractors blinded to study hypotheses identified indications for use and adverse effects through chart review. Indications other than severe allergic reactions and continuation of home use were classified as potentially inappropriate. We evaluated sex, age ≥ 85, history of cognitive impairment, drug received, and number of doses for association with adverse drug effects by regression analysis.

**Results:**

First-generation antihistamines were administered in 261 encounters (3% of geriatric ED encounters). Median patient age was 71 (range 65–107, interquartile range [IQR] 67–77) and 60.5% were female. Adverse drug effects occurred in 15% of encounters, with delirium (n = 20, 7.7%) and urinary retention (n = 11, 4.2%) being the most common. On multivariate analysis, patient age ≥ 85, history of cognitive impairment, and receipt of multiple doses were associated with elevated risk of adverse drug effects, with risk ratios of 5.5 (95% CI, 2.7–11.4), 3.1 (95% CI, 1.8–5.4), and 1.9 (95% CI, 1.1–3.6), respectively. Indications were classified as potentially inappropriate in 92% of encounters. Diphenhydramine was most used in patients with headache (n = 53, 30.1% of doses) and history of iodinated contrast media reaction (n = 46, 26.1% of doses), while hydroxyzine was most used for anxiety (n = 51, 60% of doses). The kappa value between abstractors was 0.84, indicating excellent agreement.

**Conclusion:**

Emergency department use of first-generation antihistamines in older adults, especially those **≥** 85 years of age and with prior cognitive impairment, was associated with infrequent but clinically significant harm. Most use was potentially inappropriate. Prophylactic use of diphenhydramine for patients with a prior reaction to iodinated contrast media emerged as a common indication.

## INTRODUCTION

The American Geriatric Society’s Beers Criteria identifies potentially inappropriate medications for which the risk of adverse effects may outweigh anticipated benefits for older adults. These criteria were initially created to address long-term medication prescriptions in nursing home patients and categorized medications as ones to avoid, those to use with caution, or those that should be dosed renally. In an early version of the Beers Criteria, diphenhydramine and other antihistamines were included as potentially inappropriate medications.^1^ Subsequently, this caveat was directed more specifically to first-generation antihistamines; adherence to these recommendations has been widely used as a quality measure.^2^ The criteria are intended for use in many settings, including acute care; however, there is limited evidence on the use and safety of Beers-listed medications administered to older adults during emergency department (ED) visits, in which single-dose administration predominates over long-term medication use.

Approximately 1% of all ED visits are for acute allergic reactions where antihistamines remain a part of the standard treatment regimen.^3^,^4^ The Beers Criteria recommends avoiding first-generation antihistamines in older adults except when treating severe allergic reactions due to the risk of falls, worsening delirium, urinary retention, and excessive sedation. Second-generation antihistamines are less sedating, cause fewer side effects, and are generally considered a good alternative to first-generation antihistamines in the geriatric population.^2^ We examined the use of first-generation antihistamines in geriatric patients in an urban, academic ED. We assessed the number and types of adverse drug effects, risk factors for these adverse effects, indications for use, and appropriateness of indication.

## METHODS

### Study Objective

Our primary objective was to quantify the number of patients receiving first-generation antihistamines. Secondary outcomes include indications for antihistamine use, appropriateness of indication, the number and types of adverse drug events, and risk factors for adverse drug effects.

### Study Setting and Criteria

We conducted this study at an academic ED in the Midwestern United States, using visits from January 1–December 31, 2022. During that time frame, there were 45,293 patient encounters at the ED study site, with 8,664 (19.1%) encounters for patients ≥ 65 years of age. We drafted a list of frequent indications for administration of first-generation antihistamines, which was reviewed by a geriatrician, an emergency physician, and two pharmacists. The team pre-specified anaphylaxis, angioedema, and severe allergic reactions with oral or airway involvement to be appropriate indications, as the study site does not have a parenteral second-generation antihistamines on formulary. Continuation of home medications was considered possibly appropriate, and all other indications were considered potentially inappropriate.

### Data Acquisition

We performed a retrospective cohort study of adults ≥ 65 years of age who were administered first-generation antihistamines by any route. We followed best practices for chart review research, including training abstractors with fictional records prior to data collection, using standardized abstraction forms, monitoring abstractor performance, and blinding abstractors to the study objectives until after data collection was complete.^5^–^7^ Cases were identified using the pharmacy-completed medication order function in the Epic electronic health record (EHR) (Epic Systems Corporation, Verona, WI). We developed a data abstraction form to collect the following parameters: patient age; sex; history of cognitive impairment (defined as dementia or traumatic brain injury without return to baseline documented within the EHR); a notation of which first-generation antihistamines the patient received (diphenhydramine or hydroxyzine); any adverse drug effects observed within 48 hours post-administration; and indication for use.

For inpatients, we reviewed physician and nursing notes and checked orders for possible adverse drug effects, such as an order for Foley placement. For patients released on their index visit, the EHR was reviewed to check for return visit within 48 hours. An adverse drug effect of delirium required that this be included in problem lists or diagnoses. Documentation of increased confusion without a diagnosis of delirium was counted as “confusion.”

Following abstractor training, the data abstraction form was piloted on 10 charts by each abstractor with direct supervision from the senior author (CB) to ensure accurate coding. Approximately 10% of encounters were coded by both abstractors to assess concordance of coding, and a kappa value was calculated. Charts with uncertain datapoints were flagged for review by the study team and resolved by consensus. This study was approved by the Institutional Review Board at Saint Louis University.

**Population Health Research Capsule**

What do we already know about this issue?

*Beers Criteria identify first-generation antihistamines as potentially inappropriate medications for use in older adults due to adverse drug effects*.

What was the research question?

*Among geriatric emergency department (ED) visits, what are the types of adverse drug effects associated with first-generation antihistamines, and what are the risk factors?*

What was the major finding of the study?

*Only 8.4% of indications were appropriate; age ≥85 and cognitive impairment increased the risk of adverse drug effects (P < .001)*.

How does this improve population health?

*Known risks of first-generation antihistamines are relevant to acute use in the ED, and considerable opportunity exists to decrease potentially inappropriate use*.

### Data Analysis

We compared patient sex, history of cognitive impairment, type of antihistamine, and single vs multiple doses between two subsets of patients: those 65–84 years of age; and those ≥ 85 of age. We also evaluated independent variables associated with adverse drug effects. Using chi-square tests or Fisher exact tests we calculated the crude rates of adverse drug effects in each subset and assessed the association between age and adverse drug effects events. The adjusted association of adverse drug effects events was determined using multiple logistic regression. We calculated adjusted risks of adverse drug effects in each subset using marginal probabilities based on multiple Poisson regression modeling modified with estimation of robust standard error.^8^,^9^ All statistical tests were two-tailed with an alpha at 0.05.

## RESULTS

There were 261 encounters where geriatric patients received a first-generation antihistamine (3% of all geriatric encounters). The median age of all patients was 71 (range 65–107, IQR 67–77), and 60.5% were female. Multiple doses were administered during 21 encounters, with a total of 322 doses administered. Adverse events were seen in 39 encounters, including 20 cases of delirium (20/261 7.7%), 11 cases of urinary retention (11/261 4.2%), 10 cases of excessive sedation (10/261 3.8%), two falls (2/261 0.8%), and one episode of confusion (1/261 0.4%). Five patients suffered more than one adverse drug effect (Table 1). On univariate analysis, only a prior history of cognitive impairment was associated with adverse drug effects. Indications were classified as appropriate or possibly appropriate in 22 encounters (8.4%) (Table 2).

On multivariate analysis, patients ≥ 85 years of age had an adjusted risk ratio of 5.5 (95% CI, 2.7–11.4) for adverse drug effects compared to patients < 85 (*P* < .001, Table S1). Patients with known cognitive impairment had an adjusted risk ratio of 3.1 (95% CI, 1.8–5.4) for adverse drug effects compared to those without cognitive impairment. Additionally, patients receiving multiple doses per encounter had almost twice the risk for adverse drug effects with an adjusted risk ratio of 1.9 (95% CI, 1.1–3.6). Patient’s sex and type of drug (diphenhydramine vs hydroxyzine) were not associated with adverse drug effects. The kappa was 0.84, indicating excellent agreement between the abstractors.

## DISCUSSION

In this cohort, first-generation antihistamines were administered during 3% of all geriatric patient ED visits. While the total number was low, geriatric patients continue to receive these potentially inappropriate drugs. A 2009 chart review reported that diphenhydramine was administered to 9% of hospitalized elders.^10^ Meurer et al found that diphenhydramine ranked among the top five most commonly administered potentially inappropriate medications in geriatric ED visits, and hydroxyzine among the top seven, based on a national sample from 2000–2006.^11^ In a more recent study using a national database, at least one Beers-listed potentially inappropriate medication was administered during the ED visit or prescribed on discharge for 5.9%, with diphenhydramine given in 10.1% of these visits.^12^

Indications varied by agent but were considered inappropriate on 92% of visits. Our institution approves use of first-generation antihistamines in the most severe of allergic reactions, but this is controversial. Guidelines advise against use of first-generation antihistamines for anaphylaxis due to risk of masking symptoms and delays to definitive treatment with epinephrine.^13^ Non-sedating second-generation antihistamines are considered first-line agents for urticaria and other cutaneous allergic reactions, with intravenous (IV) cetirizine being found to be non-inferior to IV diphenhydramine.^14^, ^15^

Reported iodinated contrast media allergy was the indication for first-generation antihistamines in 26% of our cohort. This was unexpected. In the most similar previous study, only 2% of diphenhydramine doses were possibly related to iodinated contrast media allergy, with the stated indication being “preprocedure (cardiac catheterization).”^16^ That study, published in 2001, included hospitalized patients; our data may be more reflective of modern ED practice. Our finding reveals a gap in guidance: prophylaxis of iodinated contrast media reaction is not directly addressed in the Beers Criteria or even the more recent, ED-specific Geriatric Emergency Medication Safety Recommendations (GEMS-Rx) criteria, which were published while our study was underway.^17^ The GEMS-Rx criteria, like the Beers Criteria, recommend against first-generation antihistamines in older adults except for “allergic reactions.” However, this allowance likely does not include iodinated contrast media reaction prophylaxis, as the recommendation is referenced to an anaphylaxis guideline that suggests against using antihistamines for iodinated contrast media reaction prophylaxis.^18^ Second-generation antihistamines may be equally effective at limiting iodinated contrast media reactions.^19^ The 2024 American College of Radiology *Manual on Contrast Media* describes diphenhydramine as “optional” and “not evidence-based” for premedication, and it allows use of a second-generation antihistamine for treatment of non-severe iodinated contrast media reactions.^20^

There are better alternatives for most other indications. The American Headache Society advises against the use of diphenhydramine for migraine treatment in the ED, regardless of age.^21^ Another common indication for first-generation antihistamines was insomnia. However, the sleep aid ramelteon is considered first-line for treatment of insomnia in elders if behavioral interventions and improvements in sleep hygiene fail.^22^

The number of adverse drug effects found in this retrospective chart review was lower than previously reported in other contexts. Prior studies suggest delirium may occur in up to 8% of hospitalized patients, and a new urinary catheter was placed in 8% of hospitalized patients receiving diphenhydramine.^16^

While the Beers Criteria are the most widely used guidelines in the US regarding medication appropriateness in older adults, not all authors agree that the Beers Criteria should be used in the ED.^23^. Hammouda et al found that 18% of elderly patients receive a prescription for potentially inappropriate medications upon discharge from the ED.^24^ Harrison et al found that 76% of geriatric patients received a new prescription for a Beers-listed medication.^25^ Neither study found an increased risk of return visits or adverse drug effects in these cohorts of discharged patients.

Future studies should explore opportunities for quality improvement initiatives promoting the use of alternative medications and to clarify the rate of adverse drug effects when substituting second-generation antihistamines. Limiting use of first-generation antihistamines in “headache cocktails” and for prophylaxis of reported iodinated contrast media reactions would appear to have the highest yield. Electronic health record alerts have been shown to reduce potentially inappropriate prescribing in elderly patients.^26^, ^27^

## LIMITATIONS

The study team pre-specified first-generation antihistamines as appropriate for treatment of severe allergic reactions given their rapid onset of action and parenteral administration, as no parenteral second-generation antihistamine was available for cases with airway involvement due to formulary restrictions at our study site. A Delphi panel of experts in geriatric emergency medicine also noted first-generation antihistamines to be appropriate in this instance.^17^

Despite our use of best practices for retrospective chart review studies, this methodology may be insensitive for detection of adverse drug effects. We searched the medical record for adverse drug effects occurring up to 48 hours after medication administration in hospitalized patients and looked for possibly related repeat ED visits within our healthcare system in the same time frame for discharged patients but may not have captured all of them. Additionally, this study did not explore drug-drug interactions or other factors that may contribute to adverse drug effects.

## CONCLUSION

We found that in a Midwestern academic ED, geriatric patients continue to receive first-generation antihistamines despite the availability of safer alternatives. We found a lower risk of adverse drug effects associated with first-generation antihistamines among geriatric patients than expected compared to existing data. However, antihistamine-associated adverse drug effects remained substantial and disproportionately affected older geriatric patients and those with pre-existing cognitive impairment. These results suggest that non-sedating second-generation antihistamines are preferable for most indications in this patient population.

## Supplementary Information



## Figures and Tables

**Table 1:** Patient demographics in a study of adverse drug effects associated with first-generation antihistamine use in older adults in the emergency department.

	All (N = 261)	Age < 85 (n = 249)	Age ≥ 85 (n = 12)	
Patient characteristics	%	%	%	*P-*value*
Female	60.5	61.0	50.0	.55
First-generation antihistamine				.76
Diphenhydramine	67.4	67.1	75.0	
Hydroxyzine	32.6	32.9	25.0	
Multiple doses	8.1	8.0	8.3	1.00
Prior cognitive impairment	14.8	14.7	16.7	.69
Adverse drug effect yes (n, %)**	39 (14.9%)	31 (12.5%)	8 (66.7%)	< .001
Delirium		16	4	
Urinary retention		8	3	
Sedation		8	2	
Fall		2	0	
Confusion		1	0	

* *P*-values reflect chi-square tests comparing frequencies by age groups. For cells with fewer than five counts, Fisher exact tests were used.

** Some patients had more than one adverse drug effect.

**Table 2:** Appropriateness of indications for first-generation antihistamine use in older adults in the emergency department.

	N (% doses)	Appropriate
Diphenhydramine		
Headache	53 (30.1%)	No
History of IV contrast reaction	46 (26.1%)	No
Pruritus	30 (17.0%)	No
Anaphylaxis	9 (5.1%)	Yes
Angioedema	7 (4.0%)	Yes
Insomnia	5 (2.8%)	No
Agitation	5 (2.8%)	No
Akathisia prophylaxis	5 (2.8%)	No
Contrast reaction	3 (1.7%)	Yes
Urticaria	1 (0.6%)	Yes
DRESS syndrome	1 (0.6%)	Yes
Other	11 (6.2%)	No
Hydroxyzine		
Anxiety	51 (60.0%)	No
Insomnia	11 (12.9%)	No
Pruritus	11 (12.9%)	No
Agitation	5 (5.8%)	No
Home medication	1 (1.2%)	Possible
Psychosis	1 (1.2%)	No
Other	5 (5.8%)	No

*DRESS*, drug reaction with eosinophilia and systemic symptoms; *IV*, intravenous.
