# Supplementary material for: First-Generation Antihistamine Use in Geriatric Emergency Department Patients: Retrospective Review
Source: West J Emerg Med. 2025 Dec 31;27(1):219–24. doi: 10.5811/westjem.47491 (PMC12815559; doi:10.5811/westjem.47491)
Supplement: Supplementary file 1 [file wjem-27-219-s001.docx]

**Table S1. Adjusted risk for ADE and risk ratios per explanatory variable**

| **Explanatory Variable** | **Comparison** | **Risk - Group 1** | **Risk - Group 2** | **Risk Ratio** | **p value** |
| --- | --- | --- | --- | --- | --- |
| **Age** | ≥ 85 vs 65 - 84 | 1.25 (0.72 - 2.17) | 0.23 (0.16 - 0.32) | 5.52 (3.03 - 10.08) | <0.0001 |
| **Cognitive impairment** | Yes vs No | 0.94 (0.56 - 1.55) | 0.30 (0.21 - 0.45) | 3.08 (1.75 - 5.41) | 0.0001 |
| **Multiple doses per encounter** | Yes vs No | 0.74 (0.43 - 1.29) | 0.38 (0.27 - 0.54) | 1.94 (1.06 - 3.56) | 0.0319 |
| **Sex** | Female vs Male | 0.48 (0.32 - 0.72) | 0.59 (0.37 - 0.95) | 0.82 (0.47 - 1.41) | 0.4694 |
| **Drug type** | Diphenhydramine vs Hydroxyzine | 0.45 (0.29 - 0.69) | 0.63 (0.40 - 1.00) | 0.71 (0.41 - 1.21) | 0.2085 |

*ADE*, adverse drug effect
